# Exercise-Based Interventions in Middle-Aged and Older Adults after Myocardial Infarction: A Systematic Review

**DOI:** 10.3390/life11090928

**Published:** 2021-09-06

**Authors:** Nebojša Trajković, Dušan Đorđević, Mima Stanković, Tanja Petrušič, Špela Bogataj, Vanja Peršič

**Affiliations:** 1 Faculty of Sport and Physical Education, University of Niš, 18000 Niš, Serbia; nele_trajce@yahoo.com (N.T.); dusandjordjevic1995@gmail.com (D.Đ.); mima.stankovic974@gmail.com (M.S.); 2 Faculty of Education, University of Ljubljana, 1000 Ljubljana, Slovenia; tanja.petrusic@pef.uni-lj.si; 3 Department of Nephrology, University Medical Centre, 1000 Ljubljana, Slovenia; spela.bogataj@kclj.si; 4 Faculty of Sport, University of Ljubljana, 1000 Ljubljana, Slovenia

**Keywords:** post-myocardial-infarction treatment, exercise, rehabilitation, physical activity

## Abstract

This study summarized the relevant literature and aimed to determine the effect of exercise-based interventions after myocardial infarction in middle-aged and older adults. Studies were identified and analyzed according to the PRISMA guidelines. The following electronic databases were used: Google Scholar, PubMed, Mendeley, Science Direct, and Scopus. The identified studies had to be longitudinal, be published in English, have taken place between 2010 and 2020, involve participants who had suffered myocardial infarction, and address different types of exercise-based interventions to be included. Of the 592 relevant studies identified, 20 were included in the qualitative analysis. After analyzing the results obtained, it could be concluded that different types of exercise-based interventions or their combination have a positive effect after myocardial infarction in middle-aged and elderly adults. It can also be suggested that the combination of a cycle ergometer and a treadmill may be the most effective exercise-based intervention. An adequate choice of intensity and volume is crucial, with the optimal duration of the experimental program and the type(s) of exercises adapted to participants after myocardial infarction.

## 1. Introduction

Myocardial infarction is an acute manifestation of coronary artery disease that affects the heart and blood vessels [1]. It is the best known cardiovascular disease that can be a major problem when it comes to the physical, psychological, and social aspects of everyone’s life [2,3], and it is currently the leading cause of morbidity and mortality [4]. This disease is associated with the formation of plaque on the inner wall of the arteries that block or reduce blood flow to the heart, resulting in damage to the heart muscle [5].

Optimal physical, mental, and social activation allows participants to regain maximum functioning. Until 1960, bed rest after myocardial infarction was considered to have the greatest health-promoting effect; however, moderate physical activity is now considered a basic therapeutic measure, as well as a preventive measure for all heart diseases [6,7]. It is a well-known and widely accepted fact that physical activity provides benefits regarding cardiovascular function, risk factor reduction, and mortality [8] regardless of age [9,10]. In addition, physical activity controls the blood pressure in people with chronic heart disease [11,12] and healthy older people [13,14], with an increase in aerobic physical activity leading to a reduction in body weight, fat percentage, hypertension [15,16], triglyceride levels, and HDL cholesterol [17]. There is also a positive effect on exercise tolerance, with increases in both exercise duration and peak power output (VO_2_peak) [18]. Participants who generally take care of their lifestyle (i.e., they do not smoke, do not consume alcohol, have an adequate exercise program with monitoring of their caloric intake) have a 54% lower risk of recurrence of myocardial infarction, and the quality of life and life expectancy automatically increases [19].

This disease can become a major challenge due to a lack of exercise [20] and increasing age if proper health monitoring is not performed. Many changes occur, ranging from the cardiovascular system [21], problems with the bone and joint system [22], and psychological changes [23], such as depression, anxiety, and decreased self-confidence. Therefore, it should be emphasized that exercise-based interventions are an important factor in strengthening the whole organism, with the response being an attempt to maintain the physiological state [24] through comprehensive adaptive processes in the respiratory, cognitive, and muscular systems [25].

The occurrence of myocardial infarction is more common in middle-aged and older populations [26]. Therefore, participants aged 18–44 years are unlikely to experience myocardial infarction compared with middle-aged (45–64 years) and older participants (≥65 years) [27].

According to the above facts and synthesis of relevant literature, the aim of this study was to determine how exercise-based interventions work after myocardial infarction in middle-aged and older adults.

## 2. Materials and Methods

### 2.1. Literature Identification

The registration number for this systematic review is 270338. Studies were searched and analyzed in accordance with the Preferred Reporting Items for Systematic Reviews and Meta-Analyses (PRISMA) guidelines [28]. PRISMA is a minimum set of evidence-based elements for reporting in systematic reviews and meta-analyses. These instructions focus primarily on helping authors to improve the reporting of their reviews by evaluating the effects of interventions, but can also be used as a basis for reporting systematic reviews with objectives other than evaluating interventions. Studies from 2010 to 2020 were included and the following databases were searched for to find appropriate and adequate literature: Google Scholar, PubMed, Mendeley, Science Direct, and Scopus.

The following keywords were used to search for the articles reporting on the exercise-based interventions in middle-aged and older adults: (“post-myocardial infarction treatment” OR “rehabilitation”) AND (“exercise” OR “physical activity” OR “high-intensity interval training” OR “medium-intensity interval training” OR “low-intensity interval training”) AND (“aerobic exercise” OR “cycle ergometer” OR “treadmill” OR “regular walking” OR “nordic walking” OR “cross-trainer” OR “rowing machine”) AND (“middle-aged” OR “adults”) AND (“older population” OR “old people”).

A descriptive method was used to analyze the data obtained, and all titles and abstracts were reviewed for possible study inclusion. At the same time, the identification strategy was modified and adapted to the particular database to increase the sensitivity. After a detailed identification process, studies were considered to be relevant if they met the inclusion criteria.

Study searches, quality assessments, data extractions, and reference lists of the available original studies were conducted and reviewed. Then, the identified studies were used for further analysis or they were discarded.

### 2.2. Inclusion Criteria

The identified studies (abstracts or whole studies) were evaluated. For the study to be included in the final analysis, it had to meet the following criteria: year of publication, the study was longitudinal, the study was published in English, the sample of participants had to be middle-aged adults (45–64 years) and older adults (≥65 years) who had suffered myocardial infarction, and the studies used different exercise-based interventions.

### 2.3. Bias Risk Assessment

The risk of bias was assessed according to the Physiotherapy Evidence Database to determine the quality of clinical trials (PEDro scale). This scale was developed to identify studies that were likely to be internally valid and have sufficient statistical information to support clinical decisions. It is a valid measure of the methodological quality of clinical trials and is a valid way to sum scale item scores to obtain the total score, which can be treated as an interval-level measure and subjected to parametric statistical analysis [29]. The results obtained from analyzing the study quality and potential risk of bias are presented in Table 1. Two independent authors assessed the quality and risk of bias using checklists. Agreement between them was assessed using the k-statistic to sift through the full text and assess the relativity and risk of bias. In the case of a disagreement about the risk of bias, data verification was performed by a third author, who also made the final decision. The k value of agreement was k = 0.94.

### 2.4. Data Extraction

After cross-examination, information was extracted and then moved to an Excel spreadsheet if the data were adequate. The Cochrane Consumer and Communication Review Group’s standardized data extraction protocol was applied to extract study characteristics, including the authors and year of study, sample size, age, experimental program types, duration, frequency, and study results.

## 3. Results

### 3.1. Study Quality

All 20 studies included in the quantitative analysis were longitudinal. Based on the points each study scored on the PEDro scale, the final study quality assessment scores were defined. With a grand total of 0–3 points, studies were classified as “poor,” 4–5 “fair,” 6–8 “good,” and 9–10 “excellent.” In addition, for studies evaluating complex interventions (e.g., exercise), a total score of 8/11 may be optimal [50]. Of all the studies included in this systematic review, only 1 study showed fair quality, 16 of them showed good quality, and the other 3 studies showed excellent quality.

### 3.2. Selection and Characteristics of Studies

**Figure 1:**
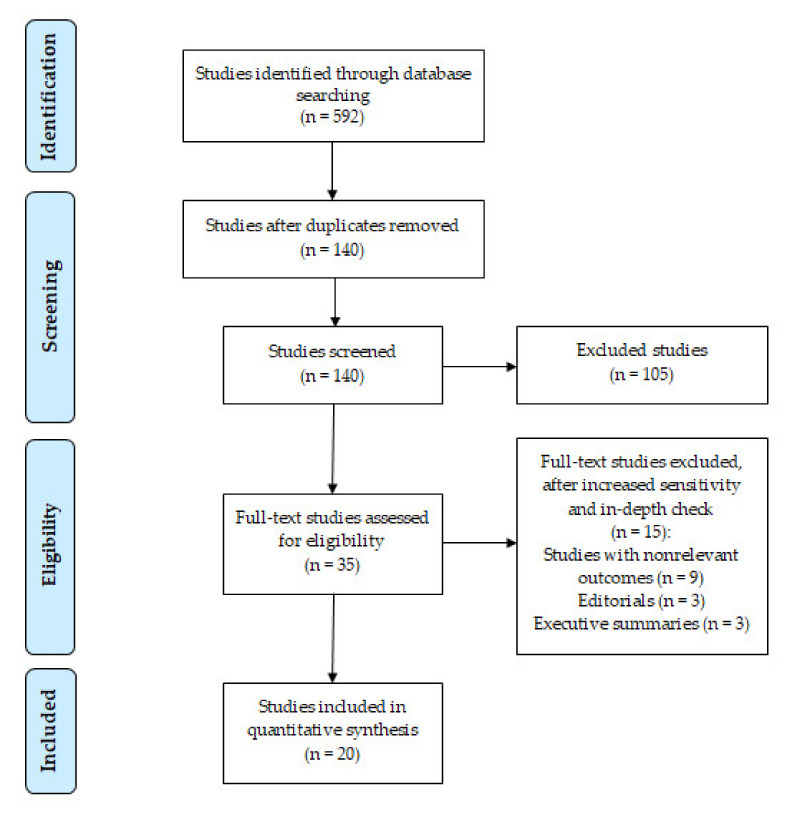
Collecting adequate studies on the basis of pre-defined criteria (PRISMA flow chart).

A search of electronic databases and scanning the reference lists yielded 592 relevant studies. After removing duplicates, 140 studies were screened. Based on the inclusion criteria, 35 studies were selected and screened for eligibility. In the end, 20 of them were included in the qualitative analysis. Figure 1 (the PRISMA flow diagram) shows the process of collecting suitable studies based on the predefined criteria.

Table 2 shows in more detail the studies that met the set conditions and entered the qualitative analysis.

There were a total of 1849 participants. The highest number was 386 [30] and the lowest was 28 [36,49]. The longest experimental program lasted 12 weeks in as many as seven studies [32,43,44,45,46,47,49], while the shortest was in the studies by Choe et al. and Balsam et al., where the experimental program lasted four weeks [34,42]. The longest training session lasted 90 min [48].

The most monitored variables were VO_2_peak [32,33,34,35,36,37,38,39,41,42,44,46,47,48,49] and resting HR [30,31,32,33,35,36,37,39,41,42,43,44,45,48,49], followed by HRpeak [30,31,32,33,34,36,44,49]. The total exercise duration (utv) was monitored in six studies [37,38,39,41,42,47], while the metabolic equivalent (METs) was monitored in seven studies [30,31,34,39,42,43,46]. Only one study did not improve the monitored variables [35].

There were studies that had only one experimental and one control group [30,31,32,33,35,37,38,41], and there were studies that included participants who all underwent an experimental intervention [34,42,43,49]. In addition, there were studies with two experimental groups [36,39,45,46,48], and there were studies that had two experimental groups with a control group [40,44,47]. In nine studies, the physical activities of the experimental program were performed on a treadmill [30,31,32,33,36,39,42,43,44]. Two studies used a cycle ergometer [34,48], while the combination of treadmill and cycle ergometer was performed in seven studies [35,38,41,45,46,47,49]. Only one study used a combination of different physical activities (treadmill, cycle ergometer, rowing machine, and cross trainer) [37]. The home program was realized in one study [40], and a combination of treadmill and home program (high-intensity interval training type) was also realized in only one study [49]. One study compared the effects of two different exercise-based interventions [32], while the correlation of certain parameters was performed by only one study [42].

Overall improvements varied from 1.1–37.2%. The greatest improvement in VO_2_peak was 22.3% [36] and these participants performed their experimental program on a treadmill, while the resting HR improved the most (10.6%) by also using a treadmill [43]. As with HRpeak, the greatest improvement was 12.6% using a cycle ergometer. The total exercise duration (utv) showed the best improvement of 19.4%, with the second-best result (16.8%) in the same study [47], where participants used a combination of a treadmill and a cycle ergometer, while the best improvement in METs (33.3%) was achieved by using the treadmill alone [30].

Most exercise-based intervention protocols consisted of aerobic training. On the other hand, there were types with high-intensity interval training (HIIT) [45,46,47,49], and also with moderate-intensity continuous exercise (MICE) [48]. Furthermore, one study compared high-intensity training (HIT) with moderate continuous training (MCT) [36]; therefore, a variety of protocols can be noted. The aforementioned training types showed improvements in VO_2_peak with better improvements presented with HIT (22.3%) versus HIIT (10.5%). The best improvement in VO_2_peak aerobic training was 21.7% [34], which can be assumed as the best effect from HIT and aerobic training. Regarding resting HR, Elshazly et al. [43] showed better improvements following aerobic training in comparison to the HIIT training presented by Dun et al. [45]. The aerobic training type [37,38,41] showed the best improvement in utv, with improvements of 15.1%, 13.2%, and 14.9%, respectively, but we should not exclude low-volume HIIT training, where the improvements were 19.4% [47]. Since Balsam et al. [34] showed the best improvements (12.6%) and Kargarfard et al. [31] (8.4%) in HRpeak, aerobic training could be suitable for the mentioned variable. Moreover, the aerobic type of training may be the best way to improve METs due to the following significant results: 33.3% by Hai et al. [30], 25.6% by Balsam et al. [34], 14.4% by Kargarfard et al. [31], 12.7% by Choe et al. [42], and 10.6% by Elshazly et al. [43]. As far as the average percentage of improvements after different types of interventions, aerobic type of training improved VO_2_peak by 12.5%, resting HR by 7.4%, HRpeak by 8%, utv by 12.7%, and METs by 19.4%. HIIT improved VO_2_peak, resting HR, and utv by 15.9%, 2%, and 1.8%, respectively. Regarding the HIT and MCT training protocols, they improved VO_2_peak by 22.3% and 9.1%, respectively; resting HR by 2% with both protocols; while utv improved by 1.9% and 2%, respectively.

As for each type of intervention program, the improvement range for treadmill use was 3–37.2%, but for a cycle ergometer, it was 6.7–35.4%. The combination of treadmill and cycle ergometer had an improvement range from 1.77 to 21.6%, while for the combination of treadmill, cycle ergometer, rowing machine, and cross trainer, this ranged from 14.6 to 16.8%. The home-based intervention [40] showed only significant improvement in submaximal functional capacity (13.3%).

## 4. Discussion

Based on a review of the relevant literature, this study aimed to determine how exercise-based interventions affect middle-aged and older adults after myocardial infarction. Physical exercise was found to provide many benefits to individuals, from preventing many non-communicable diseases and creating a better mood, to mental and emotional satisfaction, improving health, and improving physical and functional fitness [51]. Some authors believe that moderate physical activity is the basic therapeutic measure and prevention of all heart diseases, including myocardial infarction [6,7], regardless of age [9,10]. Excessive food intake, physical inactivity, and a sedentary lifestyle are associated with an increased risk of developing the disease, which is known to be more common in physically inactive people, less common in moderately active people, and least common in very physically active people [22].

In the studies by Balsam et al. and Eser et al. [34,48], using a cycle ergometer was the main physical activity. Although these two studies had no control groups, both studies show positive effects on a very wide range of variables. Balsam et al. [34] is a study that showed significant improvements in cardiorespiratory function in a very short period (12 + 12 training sessions). A nine-week study by Eser et al. [48] significantly reduced blood pressure with moderate-intensity interval training, while HIIT increased it by 4%. It should be mentioned that blood pressure variability can reduce mortality after myocardial infarction [52], and also lowering blood pressure at rest creates many benefits in subjects with cardiovascular disease [53,54,55,56,57]. Functional capacity is a well-established predictor of cardiovascular risk in elderly patients with and without a known coronary disease [58]; as such, the only way to improve it is to increase VO_2_peak [59]. Therefore, cycling an easy route can significantly improve cardiorespiratory performance in adults with lower fitness levels. The improvements may be smaller but are still significant and progressive compared to adults with intermediate and high fitness levels [60]. Since it is a non-weight-bearing activity, it puts less stress on the joints and is, therefore, less strenuous on the body [61]. It also does not require as much postural control as treadmill walking and may be a better alternative for individuals with poor balance [62].

In most studies, physical activity was performed mainly on treadmills in combination with a cycle ergometer [35,38,41,45,46,47,49]. The experimental programs lasted between 8 and 12 weeks, with an intensity of 70–95% HRmax. The combination of the abovementioned activities resulted in significant improvement in cardiorespiratory fitness, VO_2_peak, and utv, while only one study [35] showed no effects, as the subjects already showed satisfactory results at the initial measurement. Similar studies lasted longer (2–24 weeks) [63], had a slightly higher intensity [64], had an additional home program [65], and had a higher training frequency [66]. Although cycling may be a safer exercise experience, especially for participants that are unfamiliar with treadmill use, the supportive handrail on the treadmill may allow participants to achieve higher exercise intensities during exercise sessions. It is also common for various exercises to improve adherence and reduce participant boredom [67]. Therefore, it is necessary to consider the appropriate duration and adapted experimental program, the type and intensity of exercise, and the initial health status of the participants.

The experimental program of the study by McGregor et al. [37] consisted of a combination of treadmill, cycle ergometer, rowing machine, and cross-trainer. At an intensity of 60–80% VO_2_peak, there was a significant improvement of 18% in the ventilatory threshold and 16% in both VO_2_max and utv. Although exercise can be a side effect that is associated with compensatory neurohormonal mechanisms [68], it can lead to hospitalization or even death. Therefore, it is necessary to initially encourage individuals to participate in activities of daily living as a form of physical activity, with possible later progression to cardiac rehabilitation with appropriate monitoring [69]. However, both structural and functional adaptation remain fully confirmed with a combination of the above exercises. As only one study addressed the combination of these modalities, further studies are needed to confirm efficacy after myocardial infarction.

Matos-Garcia et al. [40] was the only study to examine the effects of a home walking program on respiratory strength and endurance in subjects after myocardial infarction. After two months of training, there was a statistically significant improvement in the monitored parameters. Some authors [68,70] believe that home training after cardiac rehabilitation is still a viable option, as the so-called cardiac home rehabilitation is equally effective in improving functional capacity and quality of life compared to hospital rehabilitation. The advantage of this type of intervention compared to others is the individualized treatment planning and the possibility of modification that optimizes the recovery of physical capacity without compromising the outcomes in terms of quality of life [71], functional capacity [72], and emotional status [73]. Nonetheless, it is a form of activity that can be performed in everyday life, especially by older adults [74]. This form could have public health implications, as such activity modifications can be more easily incorporated by people into their daily lives [75]. Regarding the prevalence of this disease in relation to gender, the results of the study show that the prevalence was higher in the male population than in the female population, as the total number of male subjects was 1429, which was higher than the total number of subjects, which was 1849. Only one study did not report the gender of the participants [44].

Our findings show several practical implications. However, further research is necessary to help guide exercise programs due to different populations being included in exercise programs. The best exercise-based intervention for reducing resting HR is the treadmill; for HRpeak, it is the cycle ergometer. For VO_2_peak, the best results can be obtained with the treadmill, but also with the cycle ergometer and their combination, as the results of the present study were very similar. For utv improvement, a combination of treadmill and cycle ergometer can also be used, and as for METs, a treadmill is the right choice for the best improvement. Furthermore, these results should encourage people who suffered myocardial infarction to participate in exercise-based interventions and promote exercise in different age groups, which may have an impact on poor health behaviors that are associated with this disease.

The limitation of this review is that the authors did not have complete access to all studies. In addition, a number of uncontrolled studies were found, which may influence some conclusions. A relatively small number of studies monitored caloric intake; therefore, the authors decided to group all studies under the same analysis set. In addition, no meta-analysis was conducted to provide more detailed information on which program was most effective; therefore, the authors suggest this for further studies.

## 5. Conclusions

Based on the presented results, it can be concluded that different exercise-based interventions or their combination have a beneficial effect after myocardial infarction in middle-aged and older adults. Moreover, we found that the aerobic type of intervention, i.e., the combination of a cycle ergometer and treadmill, could be the most effective exercise-based intervention. An adequate choice of intensity and volume is crucial, with the optimal duration of the experimental program and the choice of exercises being adapted to participants who suffered myocardial infarction.

## Figures and Tables

**Table 1:** PEDro scale results.

	Criterion											
Study	1	2	3	4	5	6	7	8	9	10	11	∑
Hai et al. [30]	Y	N	Y	Y	Y	Y	Y	Y	Y	Y	Y	9
Kargarfard et al. [31]	Y	Y	Y	Y	Y	N	N	Y	Y	Y	Y	8
Moholdt et al. [32]	Y	Y	Y	Y	Y	N	N	N	Y	Y	Y	7
Ribeiro et al. [33]	Y	Y	Y	Y	Y	Y	Y	N	Y	Y	Y	9
Balsam et al. [34]	Y	N	Y	N	N	N	N	Y	Y	Y	Y	5
Oliviera et al. [35]	Y	Y	Y	Y	Y	N	N	Y	Y	Y	Y	8
Kim et al. [36]	Y	Y	Y	Y	Y	N	N	Y	Y	Y	Y	8
McGregor et al. [37]	Y	N	Y	Y	N	N	N	Y	Y	Y	Y	6
Fontes-Carvalho et al. [38]	Y	Y	Y	Y	N	N	N	Y	Y	Y	Y	7
Lim et al. [39]	Y	N	Y	N	N	Y	Y	Y	Y	Y	Y	7
Matos-Garcia et al. [40]	Y	Y	Y	Y	N	Y	Y	Y	Y	Y	Y	9
Ribeiro et al. [41]	Y	Y	Y	Y	Y	N	N	Y	Y	Y	Y	8
Choe et al. [42]	Y	N	Y	Y	Y	N	N	Y	Y	Y	Y	7
Elshazly et al. [43]	Y	N	Y	Y	Y	N	N	Y	Y	Y	Y	7
Santi et al. [44]	Y	Y	Y	Y	N	N	N	Y	Y	Y	Y	7
Dun et al. [45]	Y	N	Y	Y	N	N	N	Y	Y	Y	Y	6
Dun et al. [46]	Y	N	Y	Y	N	N	N	Y	Y	Y	Y	6
Jayo-Montoya et al. [47]	Y	Y	Y	Y	Y	N	N	Y	Y	Y	Y	8
Eser et al. [48]	Y	Y	Y	Y	Y	N	N	Y	Y	Y	Y	8
Lund et al. [49]	Y	Y	Y	Y	Y	N	N	Y	Y	Y	Y	8

Legend: 1—eligibility criteria were specified; 2—subjects were randomly allocated to groups; 3—allocation was concealed; 4—the groups were similar at baseline regarding the most important prognostic indicators; 5—there was blinding of all subjects; 6—there was blinding of all therapists who administered the therapy; 7—there was blinding of all assessors who measured at least one key outcome; 8—measures of at least one key outcome were obtained from more than 80% of the subjects initially allocated to the group (dropouts); 9—all subjects for whom outcome measures were available received the treatment or control conditions as allocated or, where this was not the case, data for at least one key outcome was analyzed using “intention to treat”; 10—the results of between-group statistical comparisons are reported for at least one key outcome; 11—the study provides both point measures and measures of variability for at least one key outcome; Y—criterion is satisfied; N—criterion is not satisfied; ∑-total awarded points.

**Table 2:** Studies included in the qualitative analysis.

Study	Aim	Sample of Participants		Exercise Program	Parameters	Results
		Number and Groups	Age (Years)			
Hai et al. [30]	Evaluation of the effects in changes in heart rate recovery after exercise training in subjects after MI	N—386 E—334 C—52	E—63.8 ± 11.2 K—65 ± 10.2	2× a week 8 weeks 45 min Individual assessment of intensity Treadmill	HR HRpeak HRR METs HRpeak-HR	E ** HRpeak (121.7 ± 22.8 to 125.4 ± 23.3 bpm) + 3% HRR ** (17.5 ± 10 to 19 ± 12.3 bpm) + 8.6% METs ** (5.4 ± 3.2 to 7.2 ± 3.4) + 33.3% HRpeak-HR ** (50.5 ± 20.8 to 53.4 ± 22) + 5.74% C ** METs (4.3 ± 2.7 to 5.1 ± 3.1) + 18.6%
Kargarfard et al. [31]	Assessment of BP’s response to aerobic exercise during exercise and at rest in subjects after MI	N—72 E—35 C—37	E—57.7 ± 4.9 K—56.3 ± 5.9	3× a week 8 weeks 45–60 min 60–70% HRmax-HR Treadmill	HR HRpeak METs SBP DBP	E ** HR (79.83 ± 11.63 to 74.17 ± 10.11 bpm) − 7% * (*p* ≤ 0.001) HRpeak ** (130.93 ± 4.65 to 142 ± 3.14 bpm) + 8.4% * (*p* ≤ 0.001) METs ** (8.23 ± 1.15 to 9.42 ± 1.19) + 14.6% * (*p* ≤ 0.01) SBP ** (129.60 ± 10.97 to 123.54 ± 6.82 mmHg) − 4.7% * (*p* ≤ 0.01) DBP ** (81.43 ± 8.44 to 78.8 ± 4.34 mmHg) − 3.2% * (*p* ≤ 0.05)
Moholdt et al. [32]	Comparison of the effects of routine care and aerobic interval exercise on VO_2_peak in subjects after MI	N—89 E—30 C—59	57.4 ± 9.5	2× a week 12 weeks 60 min 85–95% HRmax Treadmill	VO_2_peak HR HRpeak HRR	E ** VO_2_peak (31.6 ± 5.8 to 36 ± 8.6 mL kg^−1^ min^−1^) + 13.9% * (*p* < 0.005) C ** HR (60.4 ± 9.3 to 56.8 ± 7.8 bpm) − 6%
Ribeiro et al. [33]	Evaluation of the effects of physical activities on the autonomic function of arterial diseases of the subjects after MI	N—38 E—20 C—18	E—54.3 ± 10.8 K—57 ± 7.6	3× a week 8 weeks 55 min 65–75% HRmax Treadmill	VO_2_peak HR HRpeak SBP DBP HRR	E ** VO_2_peak (30.8 ± 7.8 to 33.9 ± 8.3) + 10% * (*p* < 0.05) HR ** (68 ± 9.2 to 62.6 ± 8.7 bpm) − 7.9% HRR ** (20 ± 6.4 to 24 ± 4.7 bpm) + 20% * (*p* < 0.05)
Balsam et al. [34]	Evaluation of the effects of physical activity on the basic parameters of the cardiopulmonary exercise test in subjects after MI	N—52	54.1 ± 7.1	3–5× a week 12 + 12 workouts 45 min 55-65 rpm cycle ergometer	VO_2_peak HRpeak VO_2_AT VE PEF METs	VO_2_peak ** (32.3 ± 7 to 39.3 ± 12.27 mL kg^−1^ min^−1^) + 21.7% HRpeak ** (122.9 ± 16 to 138.4 ± 21.48) + 12.6% VO_2_AT ** (18.3 ± 4.4 to 24.7 ± 7.56 mL kg^−1^ min^−1^) + 35% VE ** (60.5 ± 18 to 81.9 ± 25.1 L min^−1^) + 35.4% METs ** (9.39 to 11.79) + 25.6%
Oliveira et al. [35]	Assessing the effects of cardiac rehabilitation exercise programs on HRV, assessing the effects of physical activity and dietary intake	N—92 E—47 C—45	56 ± 10	3× a week 4 weeks 50 min 70–85% HRmax Treadmill or cycle ergometer	SBP DBP HR HRV VO_2_peak	VO_2_peak ** (27.6 ± 7.3 to 29.7 ± 8.8) + 7.6% * (*p* = 0.024)
Kim et al. [36]	Assessment of the effects of HIT and MCT on VO_2_peak, as well as assessment of HIT safety in subjects after MI with an implanted stent	N—28 E_1_—14 E_2_—14	E_1_—57 ± 11.5 E_2_—60.2 ± 13.6	3× a week 6 weeks E_1_—45 min 85–95% HRR E_2_—45 min 70–85% HRR Treadmill	VO_2_max VO_2_peak HR HRpeak HRR1min	E_1_ VO_2_peak ** (29.15 ± 5.46 to 35.6 ± 7.71 mL kg^−1^ min^−1^) + 22.3%E_2_ VO_2_peak ** (27.1 ± 8.19 to 29.59 ± 8.65 mL kg^−1^ min^−1^) + 9.19% * (*p* = 0.021) E_2_ HRR1min ** (18 ± 7.4 to 23.7 ± 7.98) + 31.7%
McGregor et al. [37]	Evaluation of the effect of cardiac exercise on left ventricular structure and heart function in subjects after MI	M—50 E—33 C—17	E—55.8 ± 9.2 K—56.2 ± 10.8	2× a week 10 weeks 25–40 min 60–80% VO_2_peak Treadmill, cycle ergometer, rowing machine, cross trainers	HR BP VO_2_peak VT utv	E VO_2_peak** (24 ± 4.1 to 27.5 ± 4.6 mL kg^−1^ min^−1^) + 14.6% * (*p* < 0.05) VT ** (12.5 ± 2.8 to 14.6 ± 3.5) + 16.8% utv ** (8.6 ± 1 to 9.9 ± 1.2) + 15.1%
Fontes-Carvalho et al. [38]	Assessment of the effects of exercise on diastolic and systolic function at rest in subjects after MI	N—175 E—89 C—86	E—55.4 ± 10.3 K—55.9 ± 10.8	3× a week 8 weeks 70 min 70–85% HRmax Treadmill and cycle ergometer	VO_2_peak utv VO_2_AT BP	E VO_2_peak ** (29.1 ± 7.6 to 31 ± 9.5 mL kg^−1^ min^−1^) + 6.53% * (*p* < 0.01) utv ** (553 ± 136.1 to 625.7 ± 147.5 s) + 13.2% * (*p* < 0.01)
Lim et al. [39]	Evaluation of the effects of cardiac rehabilitation of aerobic type in both obese and non-obese subjects after MI	N—359 E_1_—170 E_2_—189	E_1_—54.3 ± 9.9 E_2_—59.1 ± 11.5	3× a week 6 weeks 50 min Individual assesment of intensity based on target HR Treadmill	HR HRmax METs VO_2_max utv	E_1_ and E_2_ HR ** (E_1_: 74 ± 1 to 70.17 ± 0.9 bpm) − 5.2% (E_2_: 77.23 ± 0.9 to 72.7 ± 0.87 bpm) − 5.9% * (*p* < 0.046)
Matos-Garcia et al. [40]	Assessment of the effects of a home-based walking program on respiratory strength and endurance in subjects after MI	N—72 E_1_—23 E_2_—31 C—18	E_1_—55.8 ± 7.5 E_2_—55.9 ± 14.6 K—48.3 ± 8.4	4× a week 60 days 35–60 min 11–12 RPE Home program	SFK	E_2_ SFK ** (43.1 ± 17.44 to 48.76 ± 13.92 cmH_2_O) + 13.3% * (*p* < 0.05)
Ribeiro et al. [41]	Assessment of the effects of aerobic exercise in cardiac rehabilitation on the level of daily physical activity in subjects after MI	N—50 E—25 C—25	E—54 ± 9 K—58 ± 9	3× a week 8 weeks 50 min 70–85% HRmax Treadmill and cycle ergometer	utv BP HR VO_2_peak	E utv ** (437 ± 204 to 502 ± 226 min) + 14.9% VO_2_peak ** (30 ± 8.1 to 32.8 ± 9.1 mL kg^−1^ min^−1^) + 9.3%
Choe et al. [42]	Relationship between %HRmax and %VO_2_max, as well as changes in the correlation of the same parameters in subjects after MI	N—66 M—54 F—12	56.7 ± 9.48	3× a week 4 weeks 50 min 60–80% VO_2_max Treadmill	HR METs utv VO_2_max HRmax	HR ** (76.3 ± 14.5 to 70.4 ± 12.1 bpm) − 7.7% METs ** (7.1 ± 1.4 to 8 ± 1.8) + 12.7% utv ** (782.3 ± 119 to 842.2 ± 146 s) + 7.7% VO_2_max ** (25.48 ± 6 to 27.86 ± 6.24 mL kg^−1^ min^−1^) + 9.3%
Elshazly et al. [43]	Assessment of the impact of physical activity on heart rate recovery in subjects after MI	N—50 M—44 F—6	51.50 ± 7.46	3× a week 12 weeks 40–60% 30min HRmax-HR Treadmill	HR HRmax HRmax-HR HRR1min HRR2min METs BP BPpeak	HR ** (76.20 ± 14.21 to 68.16 ± 8.39 bpm) − 10.6% HRmax-HR ** (58.08 ± 20.50 to 65.68 ± 16.38 bpm) + 13.1% HRR1 ** (18 ± 8.47 to 24.7 ± 7.57 bpm) + 37.2% HRR2 ** (30.52 ± 8.62 to 24.7 ± 7.57 bpm) − 19.1% METs ** (7.16 ± 1.13 to 7.92 ± 0.78) + 10.6%
Santi et al. [44]	Assessment of the impact of aerobic training at two different intensities on physical capacity and mechanical contractions of the left ventricle in subjects after MI	N—30 E_1_—10 E_2_—10 C—10	55.1 ± 8.9	3× a week 12 weeks 40 min E_1_—60–70% HRpeak E_2_—85–85% HRpeak Treadmill	VO_2_peakVEHRpeakHR	E_1_ and E_2_ ** VO_2_peak (E_1_: 19.2 ± 5.1 to 21.9 ± 5.6 mL kg^−1^ min^−1^) + 14.1% (E_2_: 18.8 ± 3.7 to 21.6 ± 4.5 mL kg^−1^ min^−1^) + 14.9%
Dun et al. [45]	Assessment of the effect of HIIT on TK and adipose tissue distribution in subjects after MI	N—120 E_1_—90 E_2_—30	E_1_—67 ± 12 E_2_—67 ± 16	3× a week 12 weeks 20–45 min E_1_—15–17 RPE E_2_—12–14 RPE Treadmill and cycle ergometer	TK HR	E_2_ TK ** BF%: 38.9 ± 6.1 to −1.9 ± 2, * (*p* < 0.001) BFM: 32.7 ± 9.2 to −1.7 ± 1.9, * (*p* < 0.001) AF%: 47.5 ± 8.1 to −2.6 ± 3.3, * (*p* < 0.001) LBM: 51 ± 10.7 to change of 1.1 ± 1.6 * (*p* < 0.01) HR ** (−4 ± 12 bpm) * (*p* < 0.003)
Dun et al. [46]	Assessment of the effect of HIIT on METs and TK in subjects after MI	N—56 E_1_—14 E_2_—42	E_1_—69 ± 14 E_2_—68 ± 10	3× a week 12 weeks 20-45 min E_1_—15–17 RPE E_2_—12–14 RPE Treadmill and cycle ergometer	TK VO_2_peak METs	E_2_ TK ** BFM: 30.9 ± 7.6 to −1.7 ± 1.8 * (*p* = 0.002) BF%: 38.8 ± 7.5 to −1.8 ± 1.7 * (*p* = 0.002) AF%: 48.1 ± 7.4 to −2.4 ± 2.4* (*p* = 0.004) LBM: 48.5 ± 8.5 to change of 1.2 ± 1.4 * (*p* = 0.01)
Jayo-Montoya et al. [47]	Assessment of changes in crf and TK using low- and high-volume HIIT programs with a Mediterranean diet in subjects after MI	N—70 E_1_—28 E_2_—28 C—14	E_1_—58.9 ± 9.6 E_2_—58.9 ± 8	2× a week 12 weeks E_1_—20–40 min 90% VO_2_peak E_2_—20 min 70% VO_2_peak Treadmill and cycle ergometer	VO_2_peak VT METpeak RERpeak utv	E_1_ and E_2_ VO_2_peak ** (E_1_: 23.1 ± 8 to 26.6 ± 7.1 mL kg^−1^ min^−1^) + 15.6% (E_2_: 23.2 ± 5.2 to 28.2 ± 7.7 mL kg^−1^ min^−1^) + 21.6% * (*p* = 0.03) METpeak ** (E_1_: 6.6 ± 2.3 to 7.6 ± 2) + 15.6% (E_2_: 6.7 ± 1.4 to 8 ± 2.3) + 19.4% * (*p* = 0.038) utv ** (E_1_: 14.3 ± 4.2 to 16.7 ± 4.7) + 16.8% (E_2_: 6.7 ± 1.4 to 8 ± 2.3 min) + 19.4% * (*p* = 0.002)
Eser et al. [48]	Assessment of acute and chronic effects of HIIT versus MICE on HR and HRV in subjects after acute MI elevation	N—69 E_1_—34 E_2_—3	E_1_—49–66 E_2_—52–62	3× a week 9 weeks 90 min E1—13–14 RPE E2—15–16 RPE Cycle ergometer	VO_2_peak HRmax HRR DBP	E_1_ DBP ** (75 to 80 mmHg) + 6.7%
Lund et al. [49]	Evaluation of the effect of HIIT on diastolic pressure in subjects after MI	N—28	56 ± 8	2× a week 12 weeks 4 × 4min 85–95% HRpeak Treadmill and cycle ergometer	VO_2_peak HR SBP DBP HRpeak	VO_2_peak ** (35.2 ± 7.5 vs 38.9 ± 7.4 mL kg^−1^ min^−1^) + 10.5%

**Legend:** N—total number of participants, M—male, F—female, MI—myocardial infarction, E—experimental group, C—control group, HRpeak—peak of the maximum heart rate, VO_2_peak—peak of maximum oxygen intake, VO_2_AT—anaerobic threshold oxygen intake, VE—minute ventilation, PEF—forced vital capacity, HRmax—maximum heart rate, VO_2_max—maximum oxygen consumption, HR—heartbeat at rest, BP—blood pressure, HRmax-HR—heart rate reserve, HRpeak-HR—heart rate gain, HRR—heart rate recovery, HRR1min—heart rate recovery after 1 min., HRR2min—heart rate recovery after 2 min., METs—metabolic equivalent (capacity), METpeak—peak metabolic equivalent, BPpeak—peak blood pressure, HIIT—high-intensity interval training, MICE—moderately intense continuous training, HRV—heart rate variability, TK—body composition, crf-cardiorespiratory fitness, ws—waist size, VT—ventilation threshold, RERpeak—the peak of the respiratory exchange relationship, SFK—submaximal functional capacity, BFM—body fat mass, BF%—body fat percentage, AF%—abdominal fat percentage, LBM—body lean mass, utv—total duration of exercise, BFM—fat body mass, SBP—systolic blood pressure, DBP—diastolic blood pressure, HIT—high interval training, MCT—moderate continuous training, rpm—revolutions per minute, RPE—rating of perceived exertion (Borg scale of 6-20), **—significant improvement, *—significant difference between groups, bpm—beats per minute.

## Data Availability

All data are already included in the main text of the manuscript.
